# Robotic intracorporeal anastomosis enhances technical precision while reducing surgeon cognitive load: a prospective multicentre study

**DOI:** 10.1007/s00464-026-13095-8

**Published:** 2026-07-09

**Authors:** Yit J. Leang, Kaleb R. Lourensz, Maria K. Vanguardia, Chrys S. Hensman, Damien Loh, Richard Chen, Andrew J. Packiyanathan, Joseph C. H. Kong, Australian Robotic Bariatric Interest Group (ARBIG), Chrys S. Hensman, Jacob A. Chisholm, Krishna P. Epari, Lilian Kow, Candice D. Silverman, Michael L. Talbot, Jason Free, Hamish Shilton, Wendy A. Brown, Paul R. Burton

**Affiliations:** 1 Department of Surgery, School of Translational Medicine, Alfred Centre, Monash University, Level 699 Commercial Road, Melbourne, VIC 3004 Australia; 2 Department of Oesophago-Gastric and Bariatric Surgery, Alfred Hospital, Melbourne, VIC Australia

**Keywords:** Robotic, Laparoscopic, Performance, Precision, Cognitive workload

## Abstract

**Background:**

Robotic surgical platforms offer enhanced dexterity and ergonomics, yet their impact on technical precision and surgeon cognitive workload (CWL) remains ill-defined. This study compared robotic versus laparoscopic gastrojejunal (GJ) anastomosis during gastric bypass to determine differences in technical precision and CWL.

**Methods:**

A prospective multicentre observational study was conducted across eight tertiary centres. Experienced bariatric surgeons beyond their learning curves performed standardised GJ anastomoses using either robotic or laparoscopic platforms. Objective performance was assessed using validated tools (BOSATS, GOALS and GEARS), and six predefined precision metrics. Surgeon CWL was measured using the NASA Task Load Index (NASA-TLX). Data were analysed using a nested mixed-effects model to control for inter-surgeon variance, with subgroup stratification by case complexity.

**Results:**

A total of 109 cases were analysed (66 laparoscopic, 43 robotic). Global objective performance scores were comparable between groups (laparoscopic 4.51 vs. robotic 4.58; *p* = 0.48). Nested analysis accounting for high inter-surgeon variance demonstrated that robotic assistance significantly reduced surgeon frustration (*p* = 0.03). Subgroup analysis revealed that the robotic platform significantly reduced overall NASA-TLX scores in both primary (*p* = 0.001) and complex revisional cases (*p* < 0.0001). Task-specific precision favoured the robotic approach in primary cases, with fewer incorrect suture placements (*p* = 0.03) and missed knot throws (*p* = 0.004). Notably, the significant inverse correlation between CWL and performance observed in laparoscopy (*r* = − 0.487, *p* = 0.001) was completely neutralized in the robotic cohort (*r* = − 0.009, *p* = 0.96).

**Conclusion:**

Robotic GJ anastomosis is associated with enhanced task-specific precision and a clinically meaningful reduction in CWL, particularly during complex revisional procedures. By decoupling surgeon stress from technical fidelity, the robotic platform acts as a performance stabilizer during demanding reconstructive tasks.

**Graphical abstract:**

**Figure:**
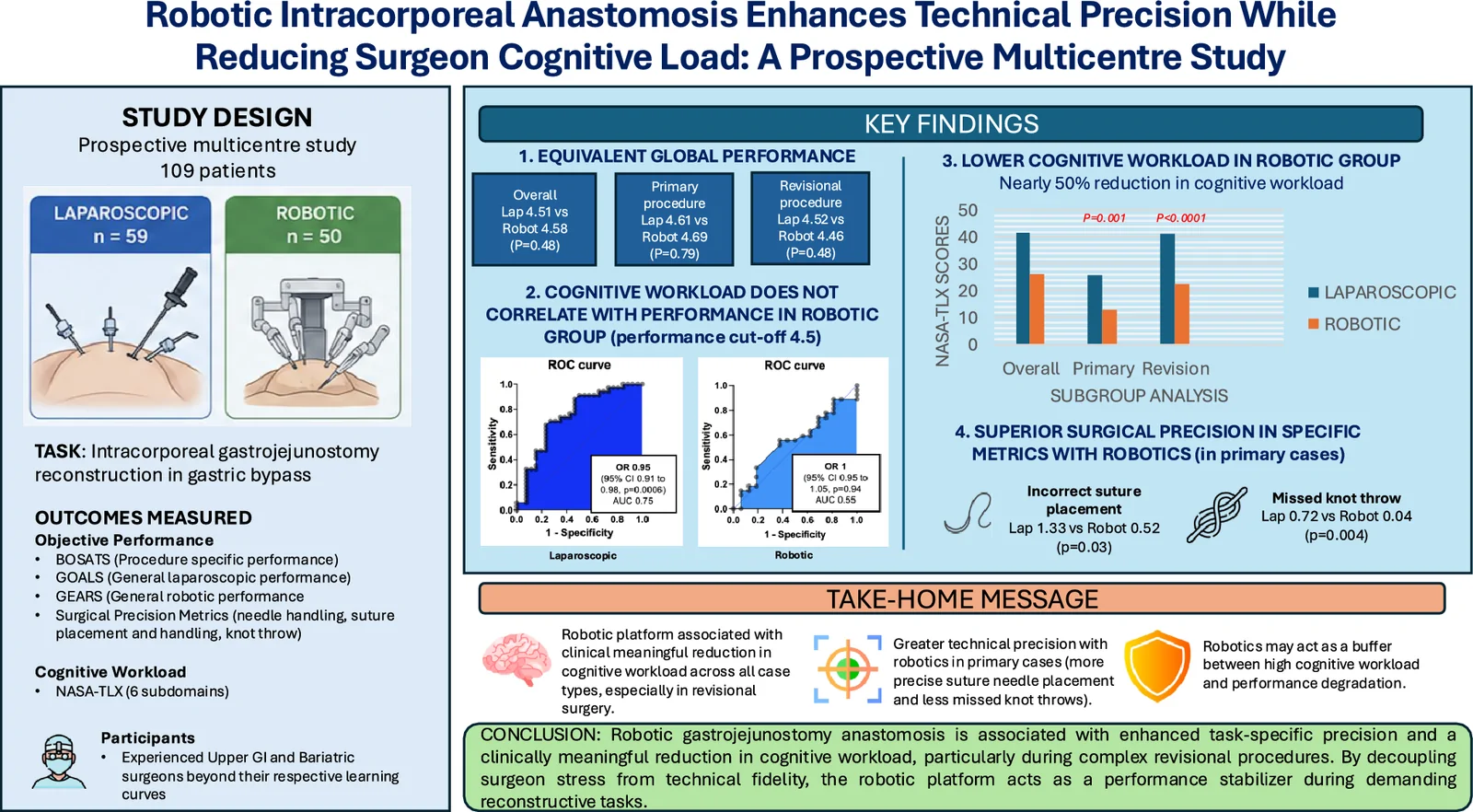

**Supplementary Information:**

The online version contains supplementary material available at https://doi.org/10.1007/s00464-026-13095-8.

Laparoscopic approach to bariatric surgery has been widely regarded as the standard of care for the surgical management of obesity over the last 2 decades [1]. However, the emergence of robotic surgical platforms has introduced a paradigm shift in technically complex minimally invasive bariatric procedures that were routinely performed laparoscopically for many years. Robotic surgical platforms offer enhanced dexterity and ergonomic advantages, yet evidence of their impact on technical precision and surgeon workload in bariatric procedures remains limited.

Robotic-assisted surgery offers several technical advantages, including articulated, wristed instruments and high definition three-dimensional visualisation. These features are hypothesized to enhance surgical dexterity, improved surgical precision during dissection and intracorporeal suturing, while potentially reducing surgeon workload [2–4]. Such benefits may be particularly advantageous in complex bariatric procedures that require the construction of gastrointestinal anastomoses, such as in Roux-en-Y gastric bypass. However, prior studies have not specifically evaluated whether robotically performed procedures offer superior technical precision in this context.

Despite these theoretical advantages, robotic-assisted bariatric surgery has yet to demonstrate significant superior clinical outcomes compared to traditional laparoscopic techniques in large-scale database studies [5–8]. This may be attributed to several confounding factors including the inclusion of cases performed by surgeons early in their robotic learning curve, variability in case complexities, and the already well-established safety profile of laparoscopic bariatric procedures.

Conventional clinical metrics such as length of stay, blood loss, and complication rates may lack the sensitivity required to detect subtle yet significant differences in surgical precision. Current comparative studies often neglect granular technical performance, despite evidence linking intraoperative surgical proficiency to clinical outcomes in bariatric surgery [9, 10]. It is plausible that robotic surgical platforms confer specific technical advantages that have yet to be systematically measured. Therefore, a necessary step in advancing the field is the rigorous evaluation of surgical proficiency as a precursor to broader assessments of clinical outcome assessments.

Beyond mechanical and physical advantages, preliminary evidence suggests that robotic surgical platforms may impose lower cognitive and physical demands on surgeons compared to conventional laparoscopic techniques. The concept of cognitive workload (CWL) and its influence on task performance has been extensively studied in high-stakes industries such as aerospace [11], military [12], and automotive engineering [13], often utilizing the validated NASA-TLX instrument [14]. These fields have effectively identified thresholds of mental demand beyond which technical performance begins to deteriorate. Applying this research framework to the high-risk, performance-critical environment of the operating theatre highlights its utility in quantifying surgeon workload and its subsequent implications for patient safety.

Given the mechanical enhancements inherent to robotic systems, we hypothesized that robotic assistance would enhance technical proficiency and reduce surgeon workload during complex manoeuvres such as construction of an intracorporeal anastomosis. Therefore, the primary aim of this study was to objectively determine if a robotically performed gastrojejunal (GJ) anastomosis offers superior technical precision and lower CWL compared to the laparoscopic approach among experienced bariatric surgeons.

## Methods

### Study design and participants

This prospective, multi-centre observational study was conducted across eight Australian tertiary hospitals from June 2023 to March 2025. The study aimed to assess the construction of the GJ anastomosis construction using either a laparoscopic or robotic platform.

Eight consultant bariatric surgeons participated in the study. All participants were highly experienced, with more than eight years of independent consultant practice. Surgeons contributing robotic cases had surpassed the initial learning curve, with a prior experience exceeding at least 100 cases as a primary console surgeon.

A consecutive enrolment strategy was employed. Surgeon and centre recruitment was ongoing after the trial commenced. Once a participating surgeon was enrolled, all consecutive eligible cases schedule to undergo elective gastric bypass procedures performed by that surgeon were recruited. Notably, one participating surgeon were not formally trained in robotic surgery and therefore contributed exclusively to the laparoscopic cohort. For each case, the intra-abdominal component of the procedure was video recorded, with specific focus on the GJ anastomosis construction for subsequent blinded assessment.

### Ethics and trial registration

The study was conducted in accordance with the Declaration of Helsinki. Written informed consent was obtained from all participating surgeons and patients for the use of intraoperative video recordings. Ethics approval was granted by the Alfred Hospital Office of Ethics and Research Governance (Approval No: 89/23), and local site-specific approvals were obtained for all participating centres. The trial was prospectively registered with the Australian and New Zealand Clinical Trials Registry (ANZCTR: ACTRN12623000086662).

### Gastrojejunal anastomosis construction video analysis

Intraoperative technical performance during GJ anastomosis construction was evaluated using three distinct modalities: a validated procedure-specific tool, a global assessment scale, and a granular technical execution metrics.

1. Procedure specific assessment: Skills were assessed using Section 9 of the Bariatric Objective Structured Assessment of Technical skills (BOSATS), which specifically focuses on the creation of the gastrojejunal anastomosis. (Appendix 1a) [15].
2. General assessment: General technical proficiency was measured using the Global Operative Assessment of Laparoscopic Skills (GOALS) (Appendix 1b) for laparoscopic cases [16] and Global Evaluative Assessment of Robotic Skills (GEARS) (Appendix 1c) for robotic cases [17].
3. Technical execution metrics: Technical precision was quantified across six predefined factors: needle drops during anastomosis, suture needle angle readjustments, incorrect suture needle placement, suture fractures, missed knot throws and frequency of camera lens cleanings.

Two independent blinded assessors graded the videos, focusing exclusively on the GJ anastomosis construction phase. Prior to the formal evaluation, the assessors participated in two 30-min calibration sessions to standardize the application of the assessment tools and ensure inter-rater reliability.

### National aeronautics and space administration: task load index (NASA-TLX)

Immediately following each procedure, the operating surgeon completed the NASA-TLX questionnaire to quantify the workload associated specifically with the construction of the GJ anastomosis. The assessment was administered via the official NASA-TLX mobile application [18]. This survey was conducted in two phases to calculate a final composite score, with higher scores reflecting greater perceived task difficulty. A comprehensive description of the NASA-TLX tool, including domain definitions and the specific calculation methodology, has been previously validated and described [19].

### Power analysis

An a priori power analysis was performed using G*Power 3.1 [20] to determine the required sample size. Given the pilot nature of this study, an anticipated effect size of 20% was assumed. With *α* = 0.05 and a power of 0.90 and based on previously reported data from the development of the Global Operative Assessment of Laparoscopic Skills (GOALS) score (mean 16.95, SD 4.75), a minimum sample size of 42 videos per group was required.

### Statistical analysis

Continuous variables were expressed as mean ± standard deviation (SD) or median and interquartile range (IQR). Inter-observer agreement was assessed using weighted Cohen’s Kappa (*κ*) coefficient. A coefficient less than 0.20 was considered slight agreement, 0.21–0.40 fair, 0.41–0.60 moderate, 0.61–0.80 substantial, and 0.81–1.00 almost perfect agreement.

Baseline patient, case characteristics and subgroup analysis (primary versus revision cases) between the laparoscopic and robotic cohorts were compared using Welch’s *t*-tests and Mann–Whitney tests as appropriate. To account for hierarchical structure of the primary outcome data, comparison of performance scores, NASA-TLX scores and technical execution metrics were performed using a nested-t test (mixed effects model) to account for baseline inter-surgeon variance. Simple logistic regression assessed the association between proficiency of the operation and NASA-TLX scores.

Two-sided *P* < 0.05 defined statistical significance. SPSS version 29 (IBM, Armonk, NY) and GraphPad Prism version 10.5.0 (GraphPad Software, Boston, Massachusetts) were used for statistical analysis and graphical illustration.

## Results

A total of 109 patients were enrolled by eight surgeons, comprising 66 laparoscopic and 43 robotic cases. All procedures were completed as planned, and video recordings for all 109 surgeries were successfully submitted for video analysis. Substantial inter-rater agreement was achieved for objective performance and technical evaluations (Cohen’s *κ* = 0.627, *p* < 0.001).

Patient demographics and case characteristics are summarized in Table 1. Both groups were comparable in terms of age, body mass index (BMI), operative weight, and sex distribution, with a high proportion of female patients in both cohorts (laparoscopic: 90.9%; robotic: 83.7%). A higher proportion of laparoscopic cases were revisional procedures (71.2%) compared to the robotic group which was more evenly divided between primary (48.8%) and revisional (51.2%) procedures (*p* = 0.04).

**Table 1:** Patient demographics and case variables

Variables	Laparoscopic	Robotic	*p*-value
Number of cases	66	43	n/a
Sex, *n* (%)			
Male	6 (9.1)	7 (16.3)	0.37
Female	60 (90.9)	36 (83.7)	
Age (years)	46.62 ± 9.92	49.85 ± 14	0.19
BMI (kg/m^2^)	39.56 ± 7.74	39.39 ± 7.58	0.91
Weight (kilogram)	102.3 (93.6–123.4)	105.6 (97–122)	0.44
Height (metre)	1.65 ± 0.08	1.69 ± 0.09	0.04
Primary	19 (28.8)	21 (48.8)	0.04
Revision	47 (71.2)	22 (51.2)	

*n/a* non applicable, *BMI* body mass index

**Fig. 1:**
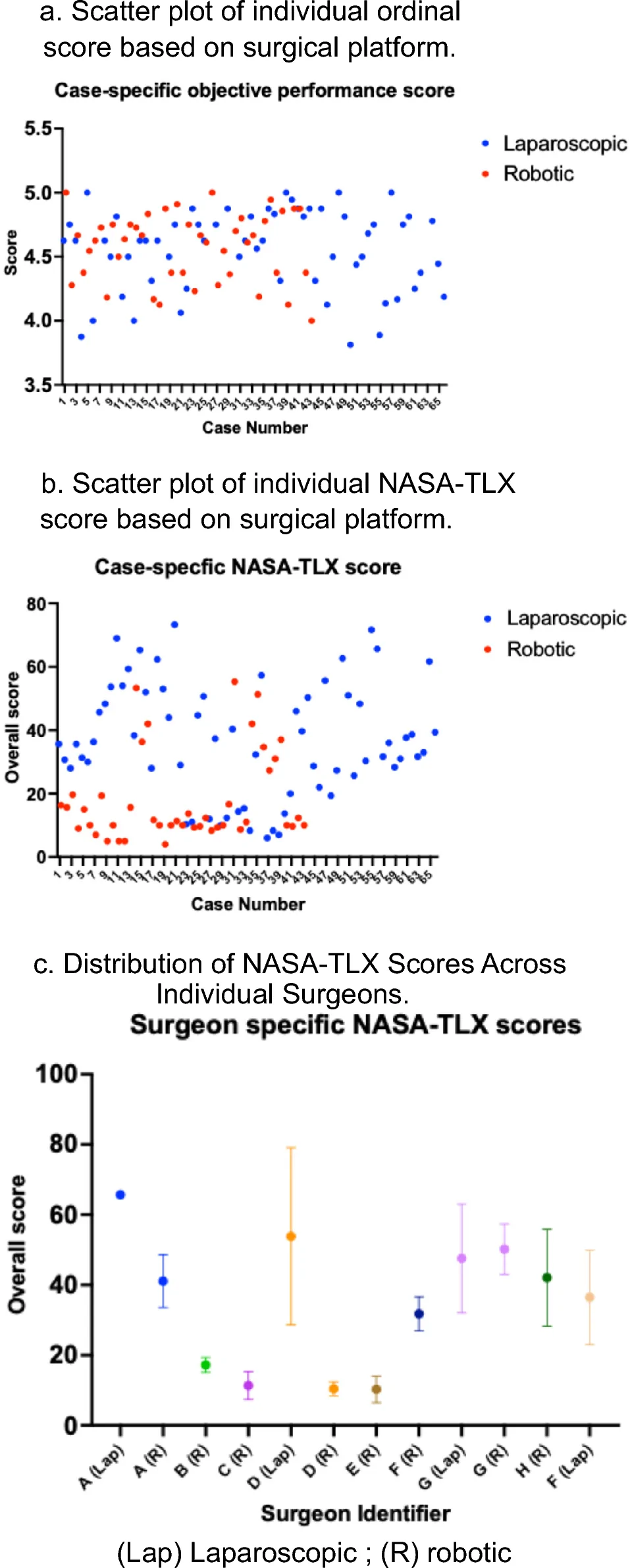
**a** Scatter plot demonstrating individual ordinal surgical performance scores stratified by operative platform (laparoscopic vs robotic). **b** Scatter plot demonstrating individual overall NASA Task Load Index (NASA-TLX) scores stratified by operative platform. **c** Mean overall NASA-TLX scores with standard deviation displayed for each individual surgeon. Surgeons A, D, and G contributed cases performed on both laparoscopic and robotic platforms

Across the entire cohort, objective surgical performance scores were consistently high and comparable between the laparoscopic and robotic groups (4.51 vs 4.58, *p* = 0.48) (Fig. 1a). Most surgeons rated the NASA-TLX scores for robotic cases under 20, with only a few outliers; notably, two robotic cases exceeded a score of 50. In contrast, nearly 50% of laparoscopic cases had NASA-TLX scores above 40, with eight cases exceeding 55 (Fig. 1b). Considerable inter-surgeon variability was observed (Fig. 1c). Surgeons A, D, F and G contributed both laparoscopic and robotic cases to the analysis. Surgeon A (Robotic: 41.08 ± 7.52; Laparoscopic: 65.67 ± 0) and Surgeon D (Robotic: 10.43 ± 1.98; Laparoscopic: 53.84 ± 25.22), reported markedly lower NASA-TLX scores for their robotic procedures. Conversely, surgeon F (Robotic: 31.78 ± 4.88; Laparoscopic 36.46 ± 13.46) and Surgeon G (Robotic: 50.22 ± 7.19; Laparoscopic: 47.56 ± 15.51) reported comparable NASA-TLX scores between the two surgical modalities.

The nested mixed model analysis revealed negligible inter-surgeon variance (variance = 0.01) for objective performance, indicating that surgeons executed the anastomosis with high technical consistency regardless of the platform or the individual operator. Evaluation of specific technical execution errors including needle drops, suture needle angle readjustments, incorrect needle placement, suture fractures, missed knot throws, and camera lens cleaning demonstrated no statistically significant differences between the two modalities across the overall cohort (all *p* > 0.05) (Table 2).

**Table 2:** Performance and execution scores

Variables	Laparoscopic	Robotic	Mean difference (95% CI)	Variance (Inter-surgeon)	*p*-value
Objective performance scores* (*maximum score: 5*)	4.51	4.58	0.07 (− 0.285 to 0.145)	0.01	0.48
Overall NASA-TLX scores	41.42	26.41	− 15.01 (− 37.54 to 7.52)	277	0.17
NASA Sub-domain scores					
Mental demand	105.2	125.1	19.93 (− 74.12 to 114)	4851	0.65
Physical demand	72.07	30.62	− 41.46 (− 108.5 to 25.57)	2369	0.19
Temporal demand	67.83	63.86	− 3.967 (− 90.98 to 83.04)	4263	0.92
Performance	162.8	86.17	− 76.66 (− 211.7 to 58.38)	9960	0.23
Effort	142.8	78.31	− 64.49 (− 144.6 to 15.61)	3278	0.10
Frustration	66.48	11.01	− 55.47 (− 105.4 to − 5.53)	613.6	**0.03**
Technical evaluation					
Needle drops during anastomosis, *n*	0.57	0.23	− 0.34 (− 0.78 to 0.11)	0	0.12
Suture needle angle readjustments, *n*	1.24	0.95	− 0.29 (− 0.99 to 0.41)	0	0.38
Incorrect placement of suture needle, *n*	1.11	1.28	0.17 (− 0.44 to 0.79)	0	0.55
Frequency of suture fracture, *n*	0.03	0.08	0.05 (− 0.05 to 0.14)	0	0.33
Missed knot throws during suturing, *n*	0.25	0.33	0.08 (− 0.20 to 0.36)	0	0.54
Frequency of camera lens clean, *n*	0.35	0.31	− 0.04 (− 0.32 to 0.24)	0	0.75

Correlation (Spearman-r) between objective performance scores and NASA-TLX scores	Spearman’s rank correlation coefficient	Confidence interval	*p*-value
Laparoscopic	− 0.487	− 0.6636 to− 0.2587	**0.001**
Robotic	− 0.009	− 0.3164 to 0.3008	0.96

Bold indicates significant *p* value <0.05

^*^Objective performance scores were derived by combining Section 9 (gastrojejunal anastomosis construction) of the Bariatric Objective Structured Assessment of Technical Skills (BOSATS) with general technical performance assessments using either GOALS or GEARS, depending on the surgical platform employed

Overall NASA-TLX scores were lower in the robotic cohort compared to the laparoscopic cohort (26.41 vs. 41.42); however, this 15-point reduction did not reach statistical significance (*p* = 0.17). The mixed model identified substantial inter-surgeon variability (variance = 277), demonstrating that individual surgeon characteristics and baseline stress levels heavily influenced overall workload independent of the surgical platform (Table 2).

Despite this high variance, analysis of the NASA-TLX sub-domains revealed that the robotic approach was associated with a statistically significant reduction in surgeon Frustration (11.01 vs. 66.48, *p* = 0.03). Other sub-domains, including mental demand, physical demand, temporal demand, performance, and effort, were lower in the robotic cohort but did not reach statistical significance in the nested overall analysis (Table 2).

**Fig. 2:**
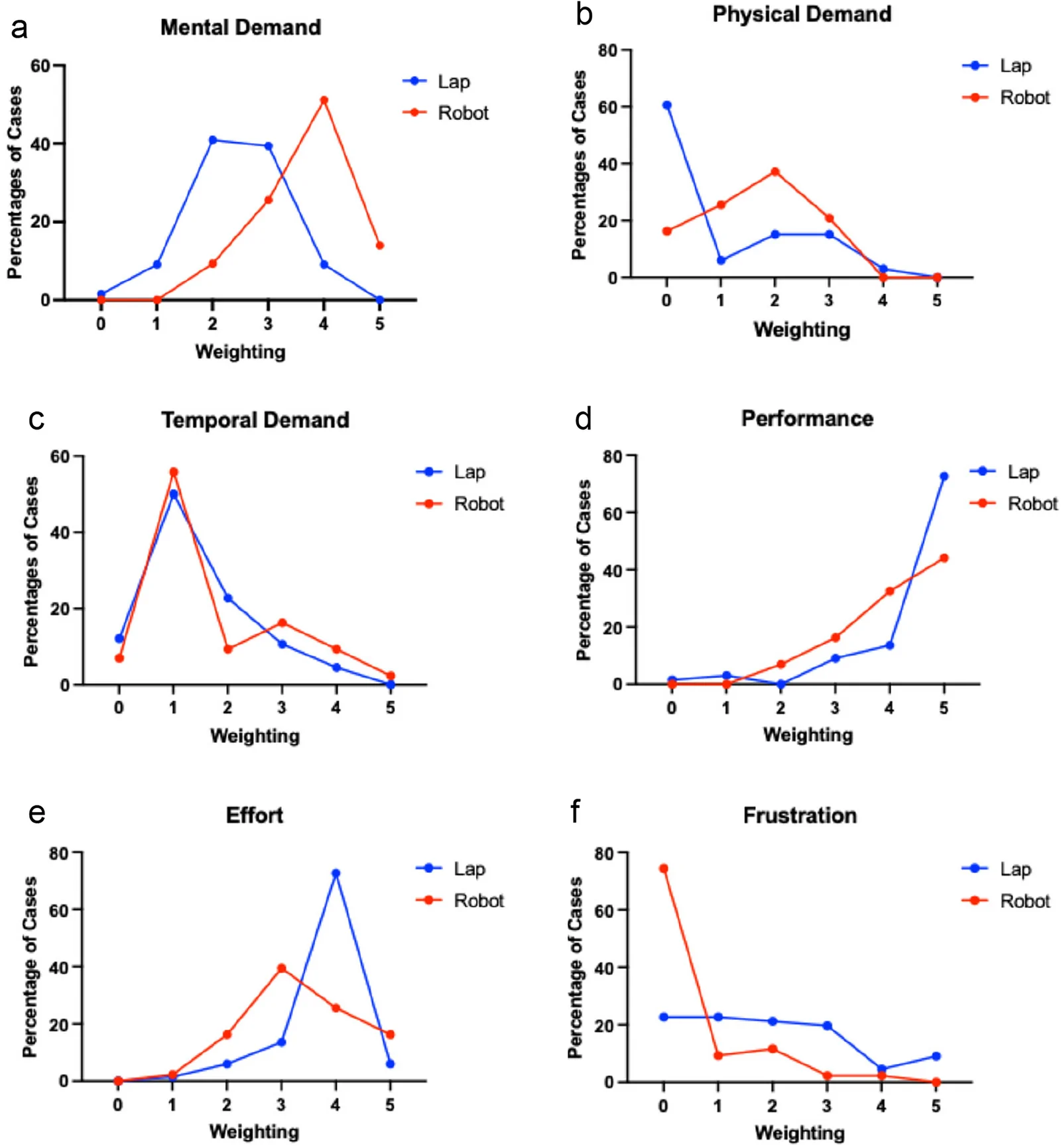
**a–f** Comparison of weighted scores across all six subdomains of the NASA Task Load Index (NASA-TLX) between the laparoscopic (Lap) and robotic (Robot) cohorts

Analysis of the relative weighting of the six NASA-TLX subdomains showed that in the laparoscopic group, performance and effort were assigned the greatest weightings. In contrast, mental demand was weighted more heavily in the robotic group. Physical demand, performance, and temporal demand demonstrated comparable weightings between the two groups, while frustration received the lowest weighting in the robotic cohort (Fig. 2a–f).

A subgroup analysis was performed stratified by primary versus revisional procedures to account for inherent differences in case complexity (Table 3), In primary cases, objective performance remained comparable between the laparoscopic and robotic cohorts (4.61 ± 0.33 vs. 4.69 ± 0.19, *p* = 0.79). However, the robotic approach yielded significantly lower overall NASA-TLX workload scores (13.10 ± 11.75 vs. 25.98 ± 17.34, *p* = 0.001) and significantly reduced temporal demand (20.95 ± 26.91 vs. 46.58 ± 54.06, *p* = 0.04). Technically, the robotic platform was associated with significantly fewer incorrect suture needle placements (0.52 ± 0.60 vs. 1.33 ± 1.24, *p* = 0.03) and missed knot throws (0.04 ± 0.22 vs. 0.72 ± 0.96, *p* = 0.004).

**Table 3:** Subgroup analysis of performance and execution scores

a. Primary procedures			
Variables	Laparoscopic	Robotic	*p*-value
Objective performance scores* *(maximum score: 5)*	4.61 ± 0.33	4.69 ± 0.19	0.79
Overall NASA-TLX scores	25.98 ± 17.34	13.10 ± 11.75	**0.001**
NASA Sub-domain scores			
Mental demand	75 ± 65.23	63.10 ± 82.51	0.29
Physical demand	53.16 ± 66.75	15.48 ± 15.16	0.17
Temporal demand	46.58 ± 54.06	20.95 ± 26.91	**0.04**
Performance	94.74 ± 92.62	39.52 ± 32.86	**0.05**
Effort	81.32 ± 60.82	49.05 ± 40.52	0.25
Frustration	37.89 ± 72.39	8.81 ± 19.87	0.08
Technical evaluation			
Needle drops during anastomosis, *n*	0	0.19 ± 0.60	0.49
Suture needle angle readjustments, *n*	1.22 ± 1.73	0.38 ± 0.74	0.06
Incorrect placement of suture needle, *n*	1.33 ± 1.24	0.52 ± 0.60	**0.03**
Frequency of suture fracture, *n*	0	0.09 ± 0.30	0.49
Missed knot throws during suturing, *n*	0.72 ± 0.96	0.04 ± 0.22	**0.004**
Frequency of camera lens clean, *n*	0.56 ± 0.86	0.14 ± 0.36	0.08

Correlation (Spearman-r) between objective performance scores and NASA-TLX scores	Spearman’s rank correlation coefficient	Confidence interval	*p*-value
Laparoscopic	− 0.415	− 0.7455 to 0.0791	0.09
Robotic	0.43	− 0.0154 to 0.7333	**0.05**

b. Revisional procedures			
Variables	Laparoscopic	Robotic	*p*-value
Objective performance scores* *(maximum score: 5)*	4.52 ± 0.30	4.46 ± 0.29	0.48
Overall NASA-TLX scores	41.15 ± 16.20	22.55 ± 14.72	** < 0.0001**
NASA Sub-domain scores			
Mental demand	67.23 ± 52.10	104.3 ± 64.92	**0.007**
Physical demand	26.38 ± 55.6	30 ± 33.70	**0.001**
Temporal demand	35.53 ± 37.78	55.91 ± 82.12	0.87
Performance	225.2 ± 109.1	71.36 ± 87.52	** < 0.0001**
Effort	176.4 ± 71.36	68.18 ± 41.71	** < 0.0001**
Frustration	86.49 ± 95.56	9.32 ± 18.73	** < 0.0001**
Technical evaluation			
Needle drops during anastomosis, *n*	0.61 ± 0.99	0.73 ± 1.45	0.58
Suture needle angle readjustments, *n*	1.32 ± 1.19	1.41 ± 2.24	0.25
Incorrect placement of suture needle, n	1.49 ± 1.49	1.09 ± 1.54	0.13
Frequency of suture fracture, *n*	0	0.14 ± 0.35	**0.04**
Missed knot throws during suturing, *n*	0.27 ± 0.59	0.18 ± 0.39	0.80
Frequency of camera lens clean, *n*	0.34 ± 0.62	0.32 ± 0.57	> 0.99

Correlation (Spearman-r) between objective performance scores and NASA-TLX scores	Spearman’s rank correlation coefficient	Confidence interval	*p*-value
Laparoscopic	− 0.350	− 0.5974 to − 0.04279	**0.023**
Robotic	0.015	− 0.4199 to 0.4448	0.946

Bold indicates significant *p* value <0.05

In revisional cases, objective performance scores again showed no significant difference (4.52 ± 0.30 vs. 4.46 ± 0.29, *p* = 0.48). However, the robotic platform demonstrated a profound ergonomic and cognitive benefit, significantly reducing overall NASA-TLX scores compared to laparoscopy (22.55 ± 14.72 vs. 41.15 ± 16.20, *p* < 0.0001). Furthermore, the robotic approach significantly mitigated almost every workload sub-domain during revisional surgery, including mental demand (*p* = 0.007), physical demand (*p* = 0.001), performance burden (*p* < 0.0001), effort (*p* < 0.0001), and frustration (*p* < 0.0001). Regarding technical execution, robotic revisional procedures were associated with a slight, but statistically significant, increase in suture fractures (0.14 ± 0.35 vs. 0, *p* = 0.04).

Overall correlation analysis identified a moderate, statistically significantly negative relationship between objective performance scores and overall NASA-TLX scores within the laparoscopic group (Spearman’s *r* = − 0.487, 95% CI: − 0.664 to − 0.259; *p* = 0.001). Conversely, such relationship was not observed in the robotic group (Spearman’s *r* = − 0.009, 95% CI: − 0.316 to 0.301; *p* = 0.96) (Table 2). Subgroup analysis by procedural complexity revealed nuanced shifts in these correlations (Table 3a and b). For laparoscopic procedures, the inverse relationship between workload and performance persisted but lost statistical significance in the primary case subgroup. Conversely, the robotic cohort exhibited a trend toward a positive correlation during primary procedures (Spearman’s *r* = 0.43, 95% CI: − 0.02 to 0.73, *p* = 0.05). Notably, this trend was completely neutralized in revisional robotic cases, where no correlation was observed (Spearman’s *r* = 0.015, 95% CI: − 0.42 to 0.44, *p* = 0.946).

**Fig. 3:**
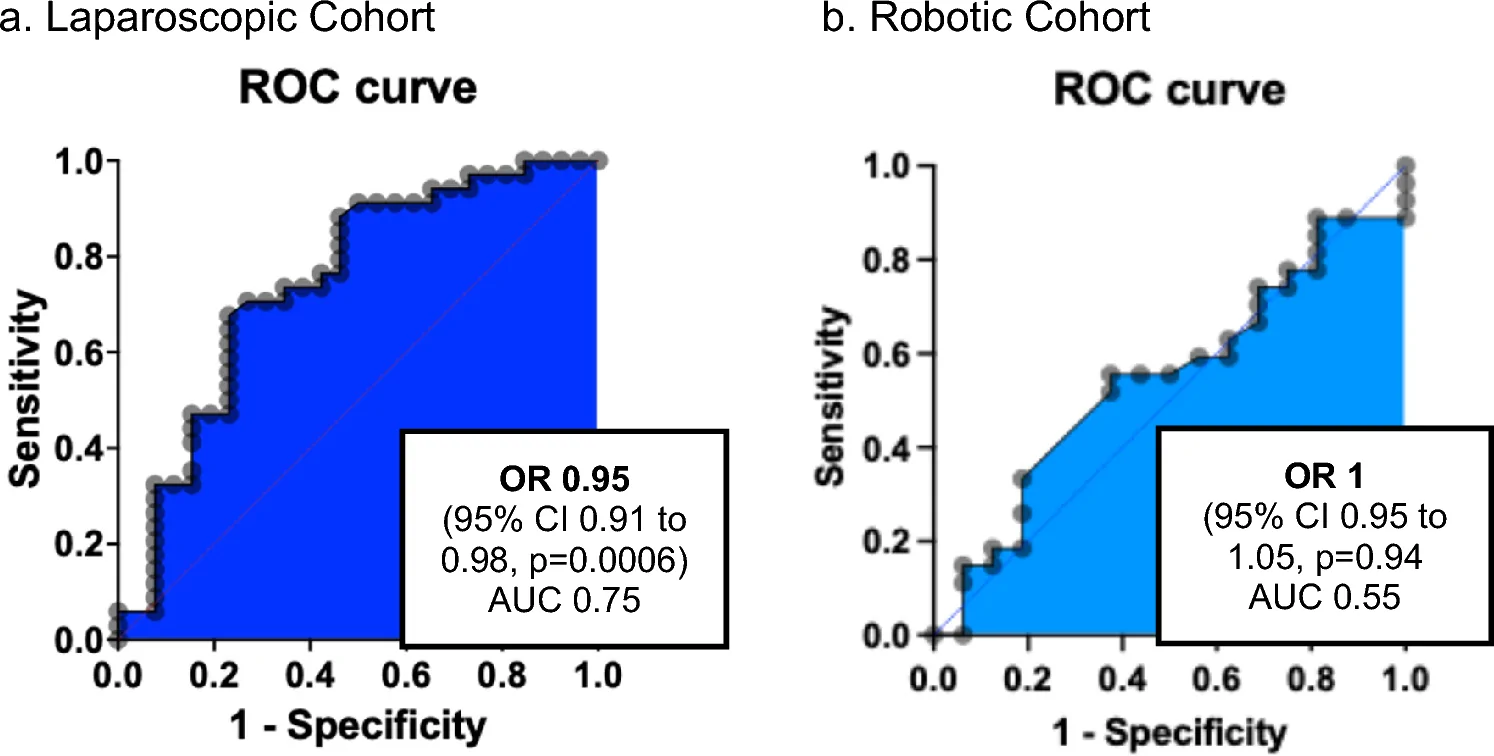
**a, b** Association between overall NASA Task Load Index (NASA-TLX) score and surgical performance using a predefined cut-off of 4.5. Cases were dichotomised into error-free and error-observed procedures and analysed using simple logistic regression

Logistic regression using a performance cut-off of 4.5 further confirmed that NASA-TLX scores were predictive of surgical proficiency in the laparoscopic group OR = 0.95, 95% CI: 0.91 to 0.98; *p* = 0.0006), but not in the robotic group (OR = 1.00, 95% CI: 0.95 to 1.05; *p* = 0.94) (Fig. 3a and 3b).

## Discussion

This prospective multicentre study of 109 patients compared technical precision and cognitive workload during intracorporeal gastrojejunal anastomosis in gastric bypass across laparoscopic and robotic platforms. Procedures were performed under real-world conditions by experienced surgeons beyond their respective learning curves. Performance was assessed using validated tools (BOSATS, GOALS, GEARS), a tailored surgical precision scale, and the NASA-TLX cognitive workload index.

While global objective performance scores (BOSATS, GOALS, and GEARS) were equally high across both groups, approaching a mean threshold of 5, the stratification of cases by baseline complexity illuminates the true ergonomic and technical value of the robotic platform. While robotic assistance reduced overall cognitive workload across all case types, the magnitude and nature of this benefit scaled directly with procedural difficulty.

During primary bypasses, the robotic approach approximately halved the overall NASA-TLX scores (13.10 vs. 25.98, *p* = 0.001). This reduction was driven predominantly by decreases in temporal demand (*p* = 0.04) and perceived performance burden (*p* = 0.05). However, as the anatomic and technical challenges escalated in revisional procedures, robotic assistance significantly mitigated mental demand (*p* = 0.007), physical demand (*p* = 0.001), effort (*p* < 0.0001), frustration (*p* < 0.0001), and performance burden (*p* < 0.0001), leaving only temporal demand unaffected. This suggests that the robot effectively absorbs the escalating physical and mental tolls inherent to revisional bariatric surgery. These findings align with recent systematic review and meta-analysis reporting consistently lower NASA-TLX scores across various robotic-assisted surgical disciplines [21].

Granular analysis of specific surgical errors revealed distinct platform-dependent profiles. In primary procedures, the robotic platform facilitated superior task-specific precision, significantly reducing the incidence of incorrect suture needle placements (*p* = 0.03) and missed knot throws (*p* = 0.004). Conversely, during revisional procedures, the robotic cohort experienced a higher incidence of suture fractures (0.14 vs. 0, *p* = 0.04). This specific technical limitation highlights a known vulnerability of modern robotic systems: the absence of haptic feedback, which becomes increasingly pronounced when managing the fibrotic, stiff, or altered tissue planes typical of revisional cases.

Perhaps the most clinically profound finding within the subgroup analysis is the shifting correlation between cognitive workload and technical fidelity. In laparoscopic revisional procedures, a significant inverse correlation was observed (*r* = − 0.350, *p* = 0.023). This suggest that as the physical and mental demands of a complex procedure increase, the surgeon’s objective technical performance reliably deteriorates.

Remarkably, the robotic platform neutralized this vulnerability during complex revisional cases, demonstrating no correlation between stress and performance (*r* = 0.015, *p* = 0.946). This phenomenon was further validated by logistic regression analysis, which showed that NASA-TLX scores significantly predicted surgical proficiency in the laparoscopic cohort (OR = 0.95; *p* = 0.0006) but failed to do so in the robotic cohort (OR = 1.00; *p* = 0.94). Together, these results suggest that the robotic platform acts as a performance stabilizer, effectively insulating the surgeon’s technical execution from the degradative effects of intraoperative stress and fatigue.

While excessive CWL is a recognized precursor to performance degradation and increased error rates in surgery, a definitive clinical threshold for these effects remains elusive [22–25]. Extrapolated data from non-surgical studies suggest that workload scores exceeding 55 correlate with significant performance decline and unforced errors; however, intraoperative surgical data characterizing such thresholds remain sparse [26, 27]. Notably, in our cohort, nearly 50% of laparoscopic cases exceeded a workload score of 40, with eight cases surpassing the 55-point threshold, whereas only two robotic cases reached such levels.

Comparative assessments of task precision between robotic and laparoscopic platforms remains limited with early investigations producing divergent findings [28]. While studies involving novice surgeons have demonstrated improved performance metrics and a subjective preference for the robotic platform [29] a large-scale study involving 117 expert surgeons previously reported superior suturing performance using the laparoscopic approach [30]. Notably, that earlier research was conducted during the nascent phase of robotic adoption, when participants possessed limited prior experience with the platform. The present study mitigated potential bias related to learning curves and platform familiarity by recruiting nationally recognized expert surgeons proficient in their respective operative modalities.

Although overall objective performance scores (BOSATS, GEARS, and GOALS) were comparably high across both platforms, this parity may reflect inherent limitations in the BOSATS scale. Originally designed for training, benchmarking, and certification, such scales may lack the sensitivity required to discriminate fine differences in surgical precision at higher levels of expert performance.

Despite the parity in global scores, the principal advantage identified in this study pertained to specific technical precision, particularly regarding suture needle manipulation and placement in primary cases. Technical analysis showed that incorrect placements were halved (0.52 ± 0.60 vs 1.33 ± 1.24; *p* = 0.03) and missed knot throws (0.72 ± 0.96 vs 0.04 ± 0.22, *p* = 0.004). This improvement is likely attributable to the enhanced dexterity afforded by wristed robotic instrumentation and its increased degrees of freedom, which facilitate more precise suture handling within the confines of a restricted intraperitoneal workspace secondary to visceral adiposity.

Current generation of robotic platforms remain limited by the absence of haptic feedback. Consequently, modulation of tissue and suture tension must be inferred solely from visual cues and surgeon experience. In our cohort, this limitation was reflected by a statistically higher incidence of suture fracture in the robotic group during revisional cases, (0.14 ± 0.35) compared to none in the laparoscopic group (*p* = 0.04). All robotic procedures in this study were performed using the da Vinci X or Xi multiport platforms (Intuitive Surgical Inc., California, USA), which do not incorporate force feedback. Several different platforms with integrated force feedback has since been introduced to address this limitation [31]. Early data suggest reductions in median force applied to tissues; however, this has not yet translated into clear improvements in clinical outcomes. Its benefits may be more evident among surgeons in training and during early skill acquisition [32, 33].

Ultimately, surgical precision remains a fundamental pursuit of operative practice and a primary determinant of favourable clinical outcomes. Although the universal adoption of robotic platforms across all surgical procedures may not be warranted, our data indicate that for experienced surgeons, the platform facilitates the construction of a demonstrably more precise intracorporeal GJ anastomosis. The identified negative correlation between CWL and technical performance in the laparoscopic cohort, which was notably absent in the robotic group, highlights the platform’s potential to insulate surgical quality from the effects of high-stress operative conditions. This "decoupling" effect suggests that the robotic platform may serve as a critical buffer, maintaining operative efficiency and precision even as subjective demand increases. Future research should focus on elucidating how these distinct workload metrics translate into tangible long-term surgical outcomes and surgeon longevity.

A key strength of this study lies was its prospective design and use of a standardised surgical task—construction of the GJ anastomosis performed by experienced surgeons beyond their respective learning curves. This approach effectively minimise variability associated with initial skill acquisition.

However, several limitations must be considered. First, although a consecutive enrolment strategy was utilized, the potential for selection bias remains. Due to resource constraints and limited access to public bariatric surgical services, most cases were recruited from privately funded medical institutions. Consequently, platform allocation was non-randomized and driven primarily by surgeon discretion, institutional workflow, and scheduling logistics. This introduces the possibility of unmeasured confounding variables, as innate surgeon preferences may have influenced platform selection in ways not captured by baseline patient demographics or surgical histories.

Second, extraneous variables such as the experience of the surgical assistant, institutional workflow demands (e.g., on-call responsibilities), and individual surgeon circumstances remain difficult to control within a clinical study design. Third, the NASA-TLX is a self-reported instrument and is inherently susceptible to individual variation in perception, memory, and cognitive bias [34]. To mitigate recall bias or the inadvertent incorporation of cognitive demands from other operative phases, the instrument was administered immediately after surgeons exited the sterile field, with explicit instructions to rate only the anastomotic reconstruction.

Finally, cognitive workload is a dynamic process; capturing transient peaks of demand remains a methodological challenge. Future research integrating multimodal, objective measures such as physiological monitoring or neuroimaging may offer more granular, real-time insights into intraoperative cognitive demand [35–37].

## Conclusion

Robotic-assisted construction of the gastrojejunal anastomosis was associated with specific improvements in technical precision compared to conventional laparoscopy, particularly in suture placement accuracy and knot-tying fidelity. While global objective performance scores remained high and comparable across both modalities, the robotic platform was associated with a clinically meaningful reduction in perceived cognitive workload especially in revisional procedures. These findings suggest that robotic systems may offer valuable ergonomic and technical advantages in complex intracorporeal anastomosis reconstruction, although further research is needed to determine how these benefits translate to long term clinical outcomes.

## Supplementary Information

Below is the link to the electronic supplementary material.

Supplementary file1 (DOCX 18 KB)

## Data Availability

The data supporting the findings of this study are available from the corresponding author upon reasonable request.
