# Supplementary material for: Robotic intracorporeal anastomosis enhances technical precision while reducing surgeon cognitive load: a prospective multicentre study
Source: Surg Endosc. 2026 Jul 9;40(9):7746–56. doi: 10.1007/s00464-026-13095-8 (PMC13582215; doi:10.1007/s00464-026-13095-8)
Supplement: Supplementary file 1 [file 464_2026_13095_MOESM1_ESM.docx]

Appendix 1

1a. Gastrojejunal anastomosis creation section of BOSATS

**Creation of gastrojejunal anastomosis - Linear stapler technique**

Create a gastrotomy in the gastric pouch

*(1-No entry into gastric lumen, poor relation between grasper and energy source, excessively large/small, penetration of posterior wall, skiving, bleeding; 3-Entry into gastric lumen, appropriate size, >1 attempt required; 5-Entry into gastric lumen, appropriate size, no extra movements required)\*

Create an enterotomy in the Roux limb

*(1-No entry into bowel lumen, poor relation between grasper and energy source, excessively large/small, penetration of posterior bowel wall; 3-Appropriate size and entry into bowel lumen, not placed in antimesenteric location; 5-Appropriate size and placement of enterotomy, good relation of grasper and energy source, no extra movements required)*

Introduce 1 limb of linear cutting stapler into gastric pouch and the other into Roux limb

*(1-Unclear how to insert the staple device, drives staple jaws blindly into the enterotomies; 3-Inserts the stapler, but lacks appreciation of the ideal angle for insertion; 5-Inserts staple jaws with ease, controlled manner, correct angle)*

Ensure both limbs are symmetrical before firing the stapler

*(1-Does not ensure symmetry, antimesenteric location of stapler before closing of jaws; 3-Limbs either nonsymmetrical or not on antimesenteric border before closure of jaws; 5-Correct symmetry and antimesenteric position before closure of jaws)*

Fire stapler

*(1-Uncontrolled fire with excessive pull on the bowel and widening of enterotomies; 3-Controlled fire, some slippage of bowel from jaws; 5-Smooth controlled fire, no widening of enterotomies)*

Suture closed the enterotomy

*(1-Poorly positioned stitch. Blindly placed sutures, traumatic use of needle drivers, skiving and poor control of needle, too much or too little tension, poor quality knot; 3-Adequate stitch or position. Adequate closure of enterotomy, sutures placed at varying distances apart, gathering of bowel edges, additional reinforcement sutures required, stitch pulled out clumsily; 5-Correct stitch position. Adequate size bites place uniform distance apart, perpendicular to seromuscular layer, appropriate tension, adequate surgical knot, needle retrieved safely)*

Ensure cinching of suture between bites

*(1-Not done; 3-Done inconsistently; 5-Done at all times)*

**Creation of gastrojejunal anastomosis - Hand-sewn technique**

Approximate Roux limb to gastric pouch end-to-end with a posterior layer of seromuscular running suture

*(1-Poorly positioned stitch. Blindly placed sutures, traumatic use of needle drivers, skiving and poor control of needle, too much or too little tension, poor quality knot; 3-Adequate stitch or position. Adequate closure of enterotomy, sutures placed at varying distances apart, gathering of bowel edges, additional reinforcement sutures required, stitch pulled out clumsily; 5-Correct stitch position. Adequate size bites place uniform distance apart, perpendicular to seromuscular layer, appropriate tension, adequate surgical knot, needle retrieved safely)*

Create enterotomy in gastric pouch and Roux limb

*(1-No entry into bowel lumen, poor relation between grasper and energy source, excessively large/small, penetration of posterior wall, skiving, bleeding; 3-Entry into bowel lumen, appropriate size, >1 attempt required; 5-Entry into bowel lumen, appropriate size, no extra movements required)\*

Place a full-thickness posterior layer running suture

*(1-Poorly positioned stitch. Blindly placed sutures, traumatic use of needle drivers, skiving and poor control of needle, too much or too little tension, poor quality knot; 3-Adequate stitch or position. Adequate closure of enterotomy, sutures placed at varying distances apart, gathering of bowel edges, additional reinforcement sutures required, stitch pulled out clumsily; 5-Correct stitch position. Adequate size bites place uniform distance apart, perpendicular to seromuscular layer, appropriate tension, adequate surgical knot, needle retrieved safely)*

Place an anterior full-thickness running suture

*(1-Poorly positioned stitch. Blindly placed sutures, traumatic use of needle drivers, skiving and poor control of needle, too much or too little tension, poor quality knot; 3-Adequate stitch or position. Adequate closure of enterotomy, sutures placed at varying distances apart, gathering of bowel edges, additional reinforcement sutures required, stitch pulled out clumsily; 5-Correct stitch position. Adequate size bites place uniform distance apart, perpendicular to seromuscular layer, appropriate tension, adequate surgical knot, needle retrieved safely)*

Insert a 34/36Fr Bougie and pass it across the anastomosis

*(1-Not performed; 3-Inserted, no guiding into correct location across anastomosis; 5-Performed correctly, atraumatic insertion)*

1b. GOALS Score

**General Rating Scale (GOALS) - Laparoscopic**

Depth perception

*(1-Constantly overshoots target, wide swings, slow to correct; 3-Some overshooting or missing of target, but quick to correct; 5-Accurately directs instruments in the correct plane to target)*

Bimanual dexterity

*(1-Uses only one hand, ignores nondominant hand, poor coordination between hands; 3-Uses both hands, but does not optimise interaction between hands; 5-Expertly uses both hands in a complimentary manner to provide optimal exposure)*

Efficiency

*(1-Uncertain, inefficient efforts, many tentative movements, constantly changing focus or persisting without progress; 3-Slow, but planned movements are reasonably organised; 5-Confident, efficient and safe conduct, maintains focus on task until it is better performed by way of an alternative approach)*

Tissue handling

*(1-Rough movements, tears tissue, injures adjacent structures, poor grasper control, grasper frequently slips; 3-Handles tissues reasonably well, minor trauma to adjacent tissue/occasional unnecessary bleeding or slipping of the grasper; 5-Handles tissues well, applies appropriate traction, negligible injury to adjacent structures)*

1c. GEARS score

Depth perception

*(1-Consistently exceeds the target, large movements, fixes slowly; 3-Some failures in making the goal, but corrected quickly; 5-Directs the instruments in the correct plane to the target)*

Bimanual skill

*(1-Use only 1 hand, ignores the non-dominant hand, poor coordination between the two; 3-Use both hands, but the interaction between them is not optimal; 5-Use both hands in a complementary manner for optimal exposure)*

Efficiency

*(1-Many tentative movements, frequent changes in the thing to do, not progress; 3-Slow movements, but organised and reasonable; 5-Confident, efficient, remains focused on the goal)*

Force control

*(1-Jerking, tearing the tissue, damage to strucutres. Frequent breaking of the suture; 3-Reasonable hnalding of tissues, less damage occurs. Occasional rupture of the suture; 5-Proper handling of tissues, proper traction thereof. Without breaking the suture)*
